# The fertility research of "Huajin 6", a new variety of honeysuckle

**DOI:** 10.1038/s41598-024-64435-4

**Published:** 2024-06-14

**Authors:** Runzhu Li, Hui Chang, Hongyan Liu, Yongqing Zhang, Conglian Liang, Gaobin Pu

**Affiliations:** 1 School of Pharmacy, Shandong University of Traditional Chinese Medicine, Jinan, 250355 Shandong China; 2 Shandong Provincial Collaborative Innovation Center for Quality Control of Traditional Chinese Medicine and Construction of the Whole Industrial Chain, Jinan, 250355 Shandong China

**Keywords:** Plant breeding, Electron microscopy

## Abstract

The aim of this study was to investigate the fertility of "Huajin 6" and the effect of exogenous methyl jasmonate on its fertility. In this study, "Huajin 6", "Huajin 6" treated with methyl jasmonate and "Damaohua" were used as the research objects, the stamen phenotypes and the shape of pollen grains were observed, pollen viability and stigma receptivity were measured. The results showed that the pistil structure and function were normal, and although the stamen anthers did not dehisce, they were still capable of producing pollen with a certain amount of vigor. Methyl jasmonate could promote the opening of the flowers of "Huajin 6" and improve the development of pollen grains to a certain extent, but it could not promote anthers dehiscence of "Huajin 6". This study can provide theoretical guidance for the cultivation of new honeysuckle varieties using "Huajin 6".

## Introduction

**Figure 1:**
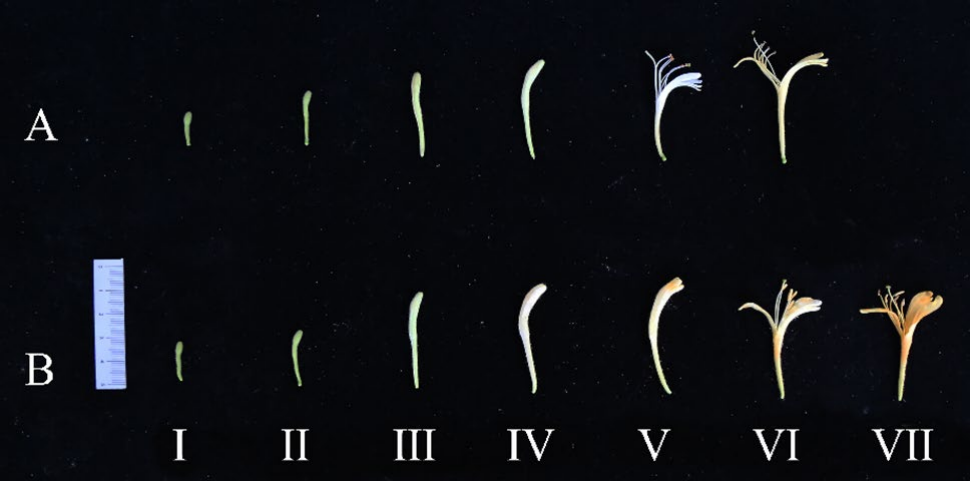
The flower buds and flowers of "Damaohua" (**A**) and "Huajin 6" (**B**) in different developmental stages. (**I**) juvenile bud stage; (**II**) third green stage; (**III**) second white stage; (**IV**) complete white stage; (**V**) silver flowering stage of "Damaohua" and dehiscence stage of "Huajin 6"; (**VI**) golden flowering stage of "Damaohua" and silver flowering stage of "Huajin 6"; (**VII**) golden flowering stage of "Huajin 6".

Lonicera japonica Flos, the dried flower buds of *Lonicera japonica* Thunb., also called honeysuckle, jinyinhua or rendong, have been used in traditional Chinese medicine to treat fever and influenza^1^, and the annual market for these dried flower buds is c. 13,000 t in China. According to the morphology of the flower buds, their development is divided into six periods, namely, the juvenile bud stage, the third green stage, the second white stage, the complete white stage, the silver flowering stage, and the gold flowering stage (Fig. 1A). "Huajin 6", a new variety of honeysuckle obtained by our research group through long-term directional breeding, is a mutant selected and bred from the "Damaohua" group that has the advantages of a long bud period and few blooms. The long budding period of "Huajin 6" can prolong and concentrate the harvesting time, greatly reduce the harvesting cost, bring new opportunities for the honeysuckle industry, and provide good materials for its breeding^2^.

"Huajin 6" has a long period of time after the complete white stage of honeysuckle, in which the petals are slightly dehisced at the top and sides but not yet fully bloomed, which is designated as the dehiscence stage in this study. Moreover, most of the corollas wilt directly after the dehiscence stage, with few blooms, and the early stage of the open flower is already partially yellowed; therefore, for the sake of comparison, the period featuring a partially white flower in the early stage of the bloom was designated the silver flowering stage of "Huajin 6" in this study, and the period with all-yellow corollas in the late stage of the bloom was designated the gold flowering stage of "Huajin 6"(Fig. 1B).

However, "Huajin 6" is characterized by a low fruiting rate in addition to its long bud phenotype, i.e. there are some defects in the fertility of "Huajin 6". Jasmonic acid phytohormones have been found to be related to plant fertility^3,4^. Jasmonic acid hormones (JAs) play key roles in the development of flowers and male organs, such as jasmonic acid (JA), which can regulate anther dehiscence in *Arabidopsis thaliana*^5^, rescue the phenotype of anther indehiscence and restore the pollen defects, cause flower elongation and seed production at *35S:AIF-C* and *AIF-C* + *SRDX* in *A. thaliana*^6^; as a JA amino acid synthase, OsJAR1 is needed for anther dehiscence in rice^7^; low levels of isoleucine jasmonate may lead to anther indehiscence in wheat^8^; and exogenous methyl jasmonate (MeJA) can reverse pollen viability in photoperiodic and heat-sensitive male sterile lines of NK19S and D01S rice^9^. The authors found that exogenous MeJA could promote the flowering of "Huajin 6", shorten its flower bud stage and greatly increase the flowering rate. Moreover, the contents of jasmonic acid phytohormones and their related synthase genes in "Huajin 6" were significantly lower than those in "Damaohua".

To identify the defects in the fertility of "Huajin 6" and to determine whether MeJA can restore its fertility, the present study was conducted to observe the stamen phenotypes, determine the stigma receptivity, detect pollen viability and observe the morphology of "Huajin 6" and MeJA-treated "Huajin 6", using the fertile variety "Damaohua" as the control, to provide theoretical support for "Huajin 6" as a cross-breeding material.

## Results

### Phenotypic observation of stamens

**Figure 2:**
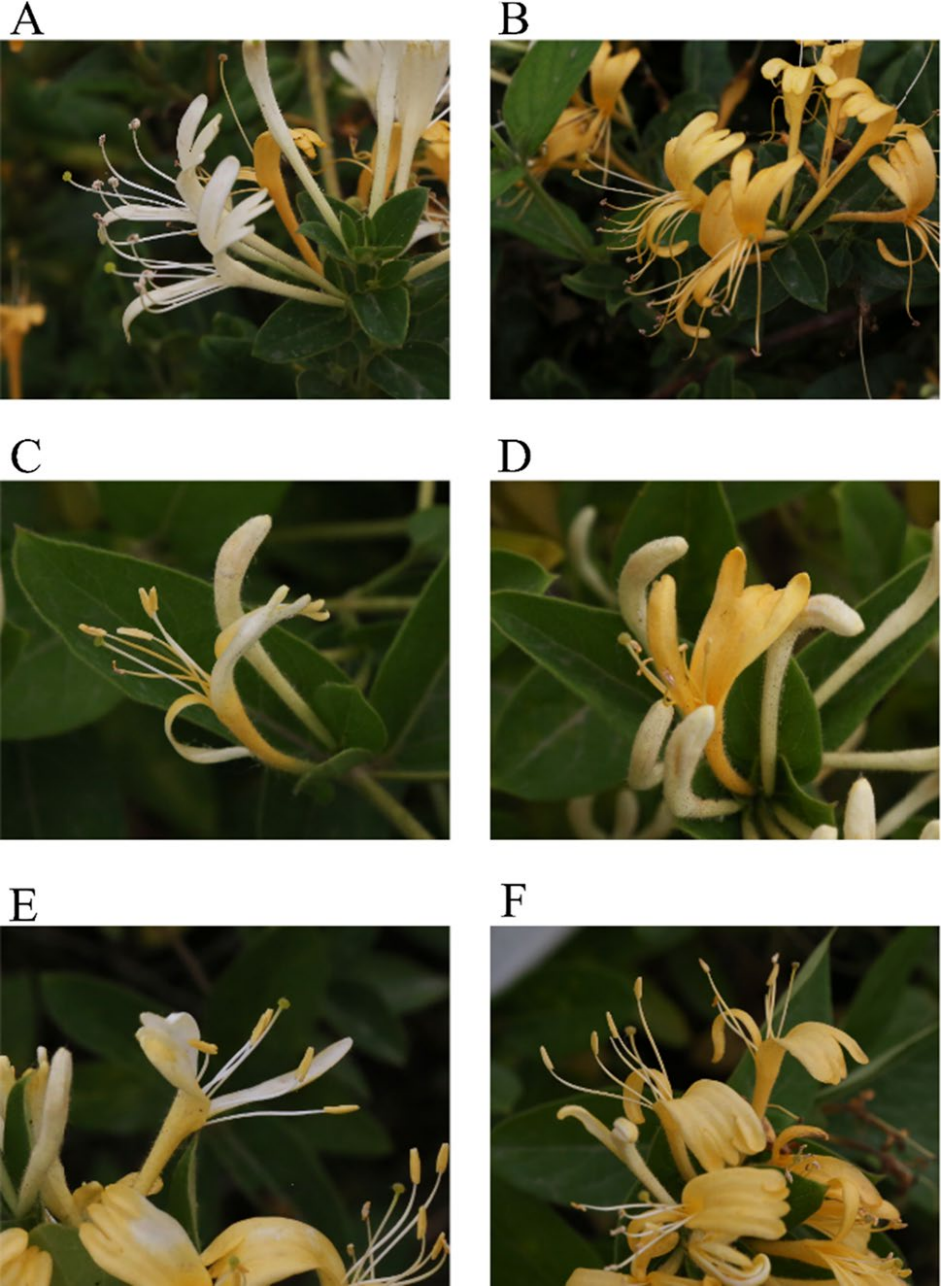
Phenotypic comparison of various varieties of honeysuckle at different periods after flowering. (**A**) The flower of "Damaohua" in silver flowering stage; (**B**) The flower of "Damaohua" in gold flowering stage; (**C**) The flower of "Huajin 6" in silver flowering stage; (**D**) The flower of "Huajin 6" in gold flowering stage; (**E**) The flower of MeJA-treated "Huajin 6" in silver flowering stage; (**F**) The flower of MeJA-treated "Huajin 6" in gold flowering stage. (**A**) to (**F**) were photographed by the author.

As shown in Fig. 2, the corolla of "Damaohua" was white at the beginning of blooming, i.e. the silver flowering stage, and the stamens and pistils protruded in a whisker-like manner and were higher than the corolla. The stigma was green with some secretion, and the anthers dehisced and dispersed a large amount of pollen (Fig. 2A). In the gold flowering stage, the stigma was yellowish-green, and the anthers were dry and brown after dispersing pollen (Fig. 2B). There were no significant differences between the appearance and development state of the pistils of "Huajin 6" and those of "Damaohua" in the silver and gold flowering stages. However, in the silver flowering stage, the anthers of "Huajin 6" were tender yellow and did not dehisce, and the dissection of the anthers revealed that a large amount of pollen was stored in the anthers (Fig. 2C). Whereas in the gold flowering stage, most of the anthers of "Huajin 6" had dried, and only a few anthers had dehisced and dispersed a small amount of pollen (Fig. 2D). The non-dehiscing anthers prevent pollen from be dispersed for pollination, resulting in the sterility of "Huajin 6". After exogenous spraying of MeJA, the flowering phenotype of "Huajin 6" recovered, there was no significant difference between the stigmas of MeJA-treated "Huajin 6" and those of "Huajin 6; moreover, the anthers in the silver and gold flowering stages were still not dehiscent (Fig. 2E and F). MeJA did not cause anther dehiscence in "Huajin 6"; in other words, MeJA could not rescue the fertility defect of "Huajin 6".

### Determination of stigma receptivity

**Figure 3:**
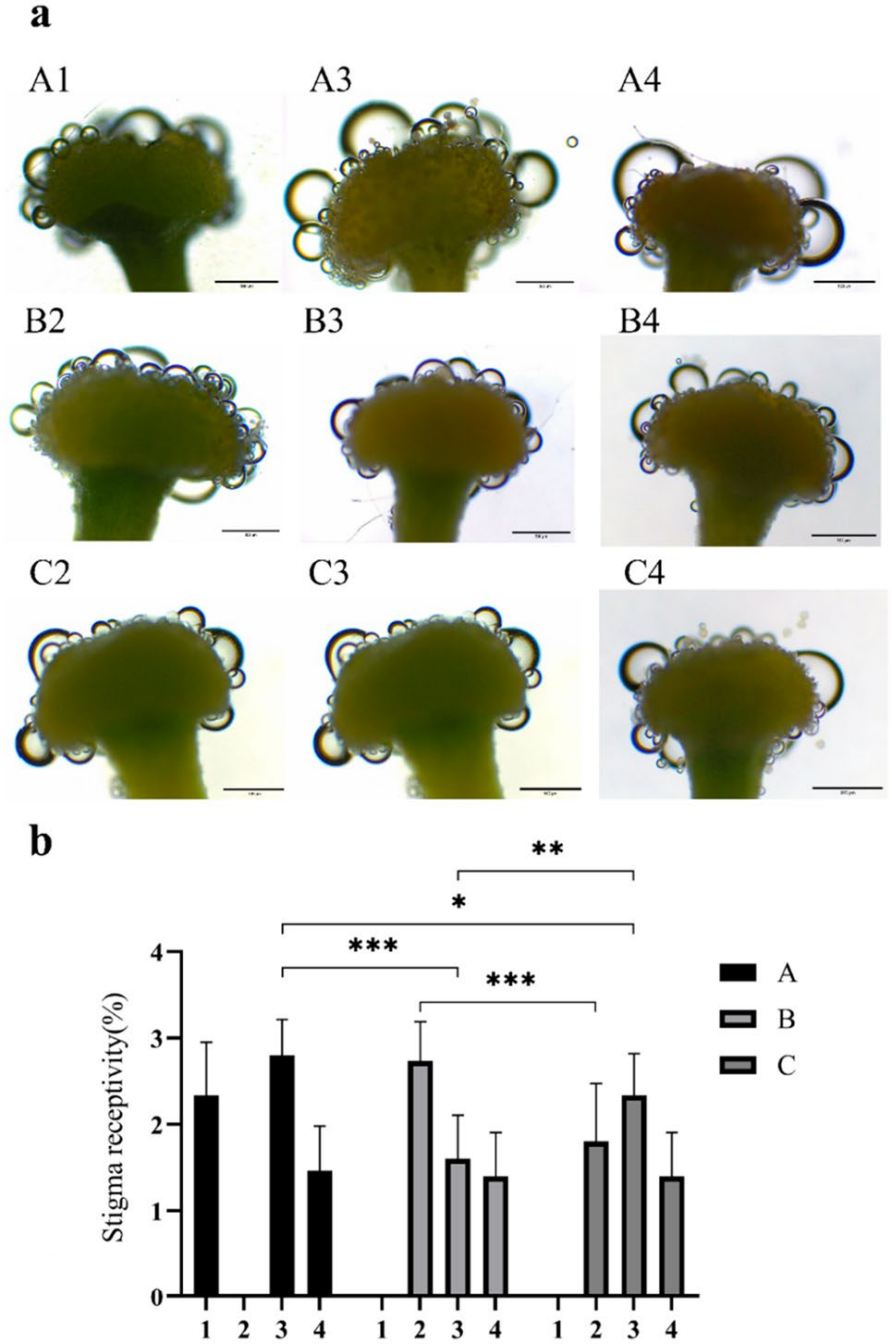
Micrographs (**a**) and statistics (**b**) of stigma receptivity at various developmental stages of different varieties. (**A**) "Damaohua"; (**B**) "Huajin 6"; (**C**) MeJA-treated "Huajin 6"; (**1**) complete white stage; (**2**): dehiscence stage; (**3**) silver flowering stage; (**4**) gold flowering stage. Error bars represent standard deviations, *p < 0.05, **p < 0.01, ***p < 0.001.

Phenotypic observation revealed that the stigma development of "Huajin 6" was normal. To verify whether the stigma function was normal, the present study compared the stigma receptivity of "Damaohua", "Huajin 6" and MeJA-treated "Huajin 6" using the benzidine-hydrogen peroxide method. According to the number of bubbles produced, the strength of stigma receptivity was divided into three grades: Grade 1 with fewer bubbles indicates less receptivity, scoring 1 point; Grade 2 with more bubbles indicates medium receptivity, scoring 2 points; Grade 3 with a large number of bubbles continuously and rapidly indicates stronger receptivity, scoring 3 points. A histogram of stigma receptivity was plotted according to the scores. As shown in Fig. 3, the stigma receptivity of "Damaohua" showed a rising and then declining trend from the complete white stage to the gold flowering stage, its stigmas produced the most bubbles in the silver flowering stage, which was the strongest in terms of receptivity; the stigma receptivity of "Huajin 6" was the strongest in the dehiscence stage, followed by the silver and gold flowering stages; the trend of stigma receptivity of MeJA-treated "Huajin 6" was the same as that of "Damaohua", and the strongest receptivity period was also the silver flowering stage. In actual breeding work, considering that the pollination of "Huajin 6" in the dehiscence stage when the stigma is most permissible needs to open the petals artificially, which is cumbersome, therefore we suggest "Huajin 6" as the female parent in crossbreeding after MeJA treatment.

**Figure 4:**
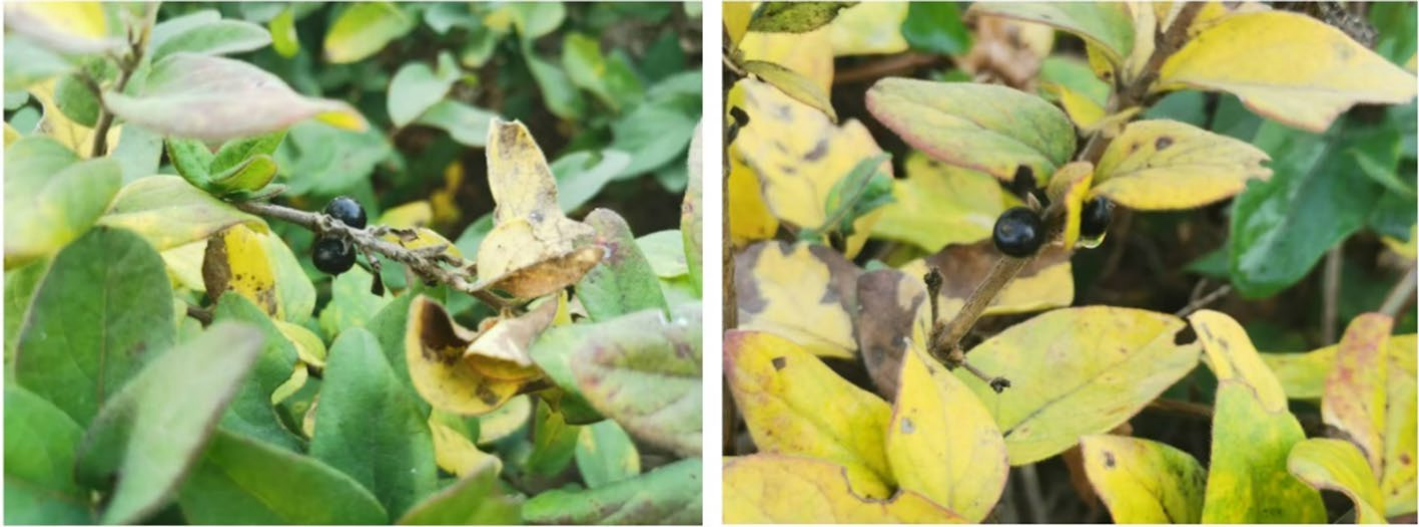
Fruits of "Huajin 6" honeysuckle hybridization experiment. Figures were photographed by the author.

The fruit setting rate was recorded on November 4, 2022, for "Huajin 6". A total of 267 flower buds were pollinated, and 204 berries were obtained. The fruit setting rate is shown in Table 1, and Fig. 4 is a photo of the fruit. The results of cross-breeding show that the fruit setting rate of "Huajin 6" can reach up to 80.494%, indicating the stigmas at the complete white stage have certain activity.

**Table 1:** Fruit setting rate of "Huajin 6" hybridization experiment.

Honeysuckle varieties	Plant number	number of fruit set	Number of pollinated buds	Mean ± s.d
Huajin6	1	58	79	73.534 ± 8.211
	2	76	95	80.494 ± 6.255
	3	70	93	75.164 ± 5.285

### Determination of pollen viability

**Figure 5:**
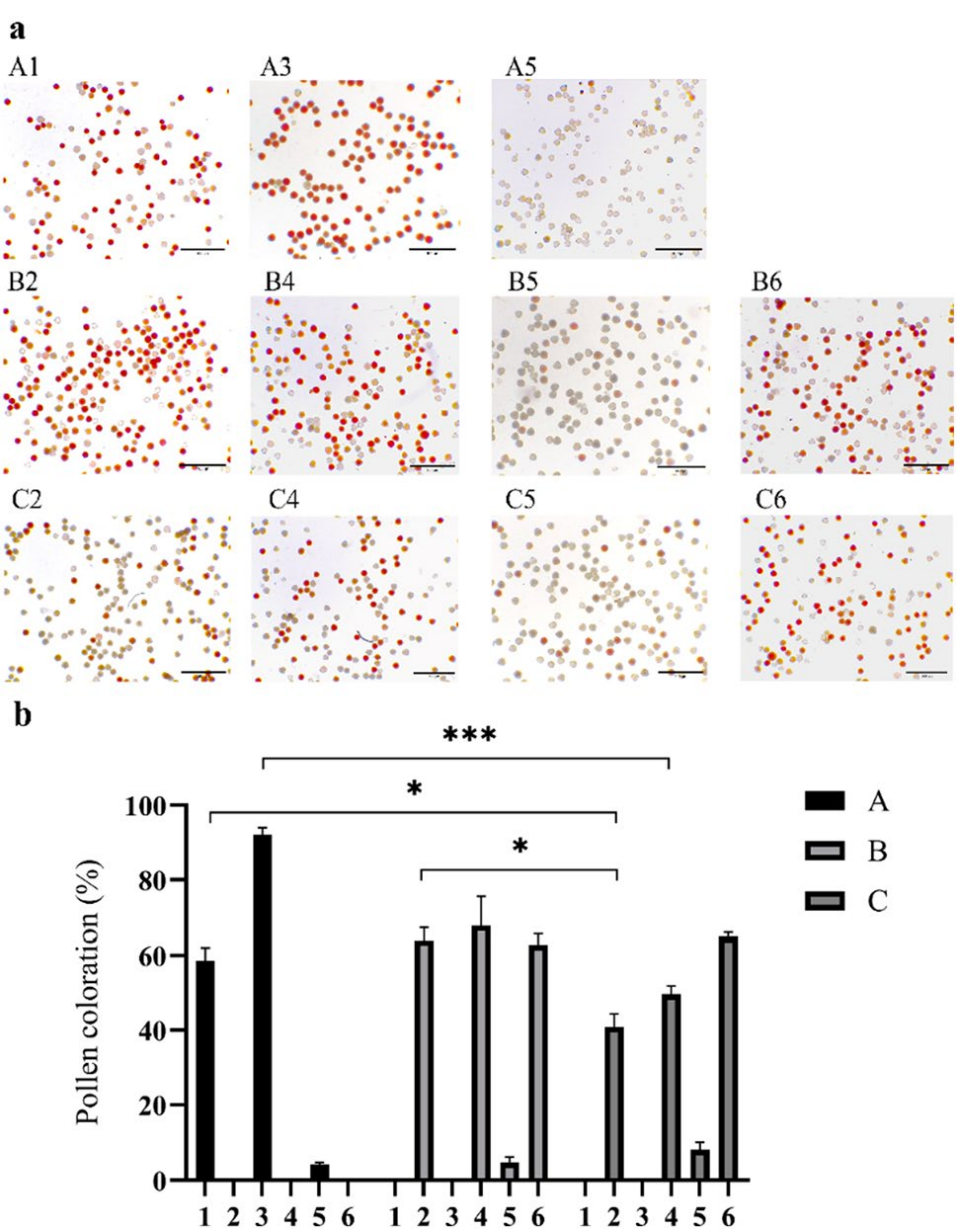
Microscopic observation of the pollen viabilities (**a**) and statistics on coloration rates (**b**) of the different varieties at different developmental stages. (**A**) "Damaohua"; (**B**) "Huajin 6"; (**C**) MeJA-treated "Huajin 6"; (**1**) complete white stage; (**2**) dehiscence stage; (**3**) silver flowering stage, pollen dispersed; (**4**) silver flowering stage, pollen not dispersed; (**5**) gold flowering stage, pollen dispersed; (**6**) gold flowering stage, pollen not dispersed. Error bars represent standard deviations, *p < 0.05, ***p < 0.001.

Although the anthers of "Huajin 6" could not dehisce, a large amount of pollen was stored in the anthers. To determine whether the pollen in the anthers was active and when it was the most active, the pollen viabilities of "Damaohua", "Huajin 6" and MeJA-treated "Huajin 6" were investigated. The stronger the pollen viability, the more easily it could be dyed red; a lighter or grayish color indicates weaker activity. From Fig. 5 and Table 2, it can be seen that the coloration rate of "Damaohua" pollen was the highest in the silver flowering stage, reaching 92.0%, while the coloration rate was only 4.3% in the gold flowering stage, at which point the pollen grains were almost inactivated. The coloration rates of the undispersed pollen of "Huajin 6" were 64.0%, 67.8% and 62.8% in the dehiscence, silver and gold flowering stages, respectively, and the pollen activity levels in the anthers before and after blooming did not change greatly, while the few pollen grains that could be dispersed were almost inactive, with a coloration rate of only 4.8%. It was hypothesized that because the anthers of "Huajin 6" had not dispersed pollen, its activity was closer to that of "Damaohua" in the unpollinated complete white stage. The pollen activity of MeJA-treated "Huajin 6" gradually increased from the dehiscence stage to the gold flowering stage, at the gold flowering stage, the pollen coloration rate was 64.8%, slightly lower than that of "Huajin 6" in the silver flowering stage; therefore, it was assumed that MeJA shortened the flower bud period of "Huajin 6" and caused it to blossom earlier than expected, but the pollen grains inside its anthers were not fully developed. After the pollen was dispersed, the pollen coloration rate of MeJA-treated "Huajin 6" decreased to 8.0%, which was higher than that of the dispersed pollen of "Huajin 6", though the pollen activity was still at a relatively low level.

**Table 2:** Pollen viability of honeysuckle in different periods.

Honeysuckle varieties	Honeysuckle development period	Pollen viability (%)			Mean ± s.d
Damaohua	Complete white stage	57.23	62.50	56.06	58.597 ± 3.431
	Silver flowering stage, pollen dispersed	91.53	90.26	94.21	92.000 ± 2.017
	Gold flowering stage, pollen dispersed	4.93	4.08	3.91	4.307 ± 0.546
Huajin 6	Dehiscence stage	64.32	60.45	67.24	64.003 ± 3.406
	Silver flowering stage, pollen not dispersed	75.78	67.44	60.09	67.770 ± 7.850
	Gold flowering stage, pollen dispersed	4.32	3.85	6.19	4.787 ± 1.238
	Gold flowering stage, pollen not dispersed	63.54	59.66	65.16	62.787 ± 2.826
MeJA-treated "Huajin 6"	Dehiscence stage	44.26	41.03	37.27	40.853 ± 3.498
	Silver flowering stage, pollen not dispersed	48.05	48.48	52.24	49.590 ± 2.305
	Gold flowering stage, pollen dispersed	6.25	10.12	7.62	7.997 ± 1.962
	Gold flowering stage, pollen not dispersed	64.12	64.15	66.23	64.833 ± 1.210

### Electron microscopy of honeysuckle pollen grains

**Figure 6:**
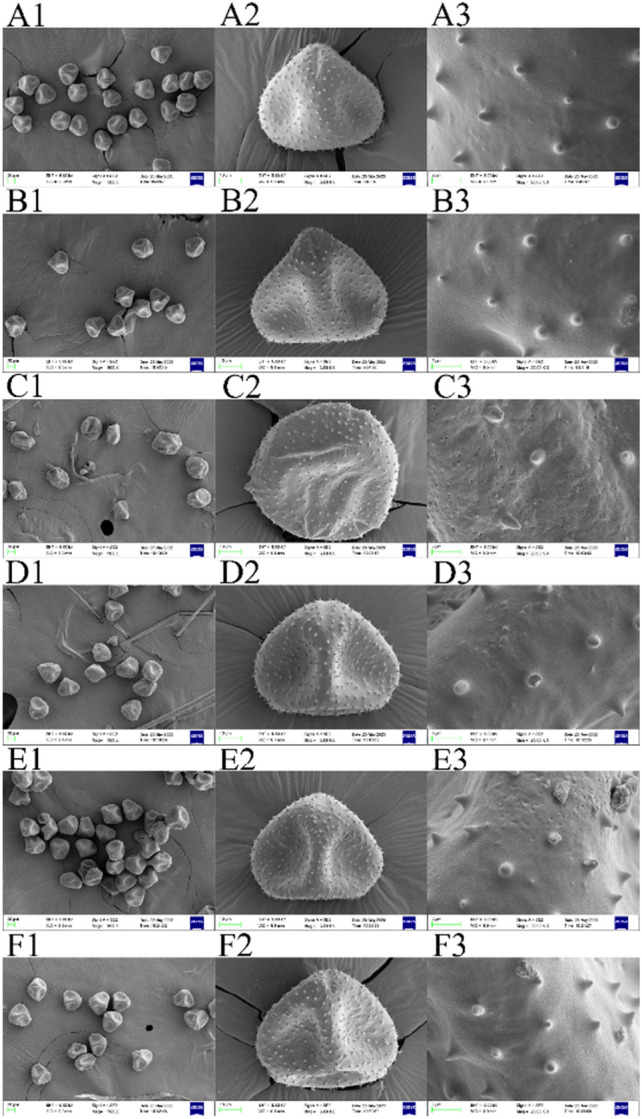
Electron microscopic observations of pollen of the different varieties. (**A**) "Damaohua" complete white stage; (**B**) "Damaohua" silver flowering stage; (**C**) "Huajin 6" complete white stage; (**D**) "Huajin 6" silver flowering stage; (**E**) MeJA-treated "Huajin 6" complete white stage; (**F**) MeJA-treated "Huajin 6" silver flowering stage; (**1**) pollen group view; (**2**) pollen grain equatorial view; (**3**) pollen grain exine ornamentation.

By observing the morphology and exine ornamentation of the pollen grains of "Damaohua", "Huajin 6" and MeJA-treated "Huajin 6", it was noted that the pollen grains of the three honeysuckle samples were all triangularly spherical with three germination pore grooves, the spiny carvings on the exine sculpturing patterns were short, blunt and uniformly distributed, and the pollen grains were all concave to different degrees (Fig. 6). Among the six samples, the pollen grains of "Damaohua" in the complete white stage were the fullest, and the pollen grains in the complete white stage and the silver flowering stage were more uniformly shaped with smooth surfaces. The pollen grains of "Huajin 6" in the complete white stage were the most wrinkled, irregularly shaped, rough and densely covered with micropores among the six samples, and the surfaces of the pollen grains in the silver flowering stage were still irregularly convex. The shape of the pollen grains of MeJA-treated "Huajin 6" in the complete white stage had been restored to a triangular spherical shape, but the surface smoothness was poor, there were still many micropores, and the surfaces of pollen grains in the silver flowering stage had no obvious convex shape and were relatively smooth.

## Discussion

As a new honeysuckle variety with excellent phenotypes, studies of the fertility of "Huajin 6" can help to cultivate new combinations featuring excellent traits by crossbreeding, such as combining "Huajin 6" with varieties with high contents of medicinal components or combining "Huajin 6" with varieties with more flowering stubble to cultivate new varieties. Hybridization in plants requires effective pollination, which requires a large amount of active pollen, an effective pollination medium, and stigmas with strong receptivity^10,11^. In this study, we investigated the pollen viability and stigma receptivity of "Huajin 6". Phenotypic observation revealed that the stigma of "Huajin 6" did not differ much from that of "Damaohua", and MeJA had little effect on the stigmas; however, the stamen anthers of "Huajin 6" did not dehisce. Anther dehiscence, the last stage of anther development, determines whether a flower can be pollinated properly and is a key step in plant development^12^. If the anthers do not dehisce but pollen can be produced inside the anthers and the pollen can develop to maturity, this represents the functional male sterile phenotype^13^. Dissecting the anthers of "Huajin 6" revealed that there was a lot of pollen inside; therefore, it was initially hypothesized that "Huajin 6" was a functionally male sterile line, and MeJA could not restore its natural fertility.

The results of the stigmatic pollination study revealed that the stigma receptivity of the normal-fruiting honeysuckle variety "Damaohua" was strongest in the silver flowering stage, while the stigma receptivity of "Huajin 6" was strongest in the dehiscence stage, as evidenced by data from hybridization experiments, after which receptivity gradually decreased. After spraying MeJA, "Huajin 6" recovered its flowering phenotype, and the stigma receptivity was strongest in the silver flowering stage. It is assumed that the pistil development time of "Huajin 6" is the same as that of the normal-fruiting "Damaohua", but due to its long-bud stage phenotype, the stigmas would be less fertile when the petals opened, making it unsuitable for pollination; MeJA did not affect the development of the pistils. The results of this study suggest that we can use "Huajin 6" as the female parent for crossbreeding directly after MeJA treatment.

The results of the pollen viability study indicate that the pollen viability of the honeysuckle variety "Damaohua", which can bear fruits normally, peaks in the silver flowering stage, while the stigma also developes and matures in this period and is the most receptive; at this time, "Damaohua" blooms and completes its pollination. The undehisced anthers of "Huajin 6" can produce active pollen, indicating that "Huajin 6" is indeed a functional male sterile line. It has been found that MeJA can promote anther of sterile alfalfa lines to dehisce^14^. This study revealed that MeJA could restore the flowering phenotype but did not cause anther dehiscence, and the pollen viability of MeJA-treated "Huajin 6" anthers showed an increasing trend from the dehiscence stage to the gold flowering stage. The strongest activity was observed in the gold flowering stage, and the pollen viability level was similar to that of "Huajin 6", indicating that MeJA was able to promote flowering but did not increase the pollen activity. The pollen grains of sterile male onion lines are often characterized by deformation, collapse, and nonsmooth surfaces^15^; in the study of the *pwa1* mutant of a rice male sterile line, it was found that its sterile pollen was associated with abnormally developed pollen walls^16^. The surfaces of pollen can indicate the maturity of pollen grains and thus play an important role in maintaining the structure and function of pollen and can directly affect the fertility of the pollen grains. Electron microscopic visualization of the pollen grains revealed that MeJA could promote the development of pollen grains in "Huajin 6" to a certain extent.

## Materials and methods

### Materials

The test materials were "Huajin 6" and "Damaohua", which were planted in the Garden of Medicinal Plants of Shandong University of Traditional Chinese Medicine. The flowers and buds of "Huajin 6" and "Damaohua" were collected in May 2023 at the complete white, dehiscence, silver flowering and gold flowering stages. The flowers and buds collected from 8:00 a.m. to 9:00 a.m. on the same day were used for determinations of pollen viability and stigma receptivity; the pollen shade-dried was used for electron microscopic observation.

### Methods

#### Methyl jasmonate treatment

A 400 μmol·L^–1^ MeJA solution was prepared as the treatment liquor. It was sprayed on three "Huajin 6" plants with the same growth conditions every 3 days, starting from the juvenile bud stage.

#### Determination of pollen viability

Pollen viability was determined by 2,3,4-triphenyltetrazolium chloride (TTC) staining. Five buds or flowers were harvested from three honeysuckle plants with similar growth in each group, and an appropriate amount of fresh pollen was collected and placed in a centrifuge tube, and 1 mL of 0.5% TTC solution was added, mixed well, and then placed in a 37 °C incubator for 20 min, followed by mixing with a pipette and aspirating onto a slide, after which the staining was observed under a low-magnification microscope. Three mounts were made for each group of samples, and five fields of view were observed for each mount. Pollen coloration rate = number of red pollen grains/total number of pollen grains × 100%^17–19^.

#### Determination of stigma receptivity

The benzidine-hydrogen peroxide method was selected to test stigma receptivity. The stigmas were placed on slides, and an appropriate amount of benzidine-hydrogen peroxide solution (1% benzidine: 3% hydrogen peroxide: water, v/v/v = 4:11:22) was added dropwise so that each stigma was completely submerged, and the number of bubbles was observed under a low-magnification microscope^20–23^.Three honeysuckle plants were observed in each group, and the stigmas of five flowers from each honeysuckle plant were observed。

In addition, our group conductied cross-breeding experiments using "Damaohua" as the paternal parent and "Huajin 6" as the maternal parent. On May 12, 2022, three "Huajin 6" plants with the same growth were selected, with five inflorescences for each plant. Only the complete white stage bud was retained, and the stamen and upper half of the petals of the bud were removed before placing the breeding bag over them. The fruit setting rate was observed six months later.

#### Scanning electron microscopy of pollen grains

A small amount of shade-dried pollen was dipped with a pollination pen and stuck evenly on an electron microscope sample preparation stage. After being sprayed with gold, it was visualized by electron microscopy.

#### Statistical analysis

Significance analyses were performed using the two-way ANOVA function in GraphPad prism 8.0 software, and graphing was performed via GraphPad prism 8.0 software.

### Statement

All methods in regards with plants were carried out in accordance with relevant guidelines in the method section and plants were collected with proper permits.

## Data Availability

The datasets generated during and/or analysed during the current study are available from the corresponding author on reasonable request.
